# Mental health, COVID-19 burden and quality of life of kidney transplant recipients two years after the COVID-19 pandemic

**DOI:** 10.3389/fpsyt.2024.1338934

**Published:** 2024-05-01

**Authors:** Concetta De Pasquale, Maria Luisa Pistorio, Massimiliano Veroux, Noemi Barbagallo, Provvidenza Marisa Cottone, Burcin Ekser, Giuseppina Lorenzano, Alessia Giaquinta, Pierfrancesco Veroux

**Affiliations:** ^1^ Vascular Surgery and Organ Transplant Unit, Department of Educational Sciences, University of Catania, Catania, Italy; ^2^ Vascular Surgery and Organ Transplant Unit, Department of General Surgery and Medical-Surgical Specialties, University Hospital of Catania, Catania, Italy; ^3^ Organ Transplant Unit, Department of Surgical and Medical Sciences and Advanced Technologies, University Hospital of Catania, Catania, Italy; ^4^ Regional Transplant Center, Department of Advanced Diagnostics and Services, Civico and Di Cristina Hospital, Palermo, Italy; ^5^ Department of Surgery, Indiana University School of Medicine, Indianapolis, IN, United States

**Keywords:** kidney transplantation, mental health, COVID-19, psychopathology, quality of life

## Abstract

**Introduction:**

Few studies have evaluated the psychological distress of COVID-19 in kidney transplantation and the psychological impact that the COVID-19 pandemic has had on kidney transplant recipients is not yet well understood. The present study aimed to investigate the change in symptom burden and health-related quality of life in the two years after initial assessment, by outlining the change over time of symptoms at 12 and 24 months of follow-up.

**Methods:**

This is a follow-up study. We performed a study published in 2021 (phase 1 of COVID-19); of the 89 kidney transplant recipients evaluated in this study, 60 completed the 12 months follow-up (March 2021 June 2021, phase 2 of COVID-19) and 57 completed the 24 months follow-up (March 2022 June 2022, post COVID-19). The same tools as in previous study were administered: the *ad hoc* questionnaire on emotional state and psychophysical well-being during COVID-19, the Middlesex Hospital Questionnaire (MHQ) to provide a simple and rapid quantification of the psychological and somatic symptoms and the Short Form Health Survey 36 (SF-36) was used to assess health-related quality of life.

**Results:**

Compared to the first and second phase of COVID-19, the mean score of quality of life variables were higher in the post COVID-19 phase; thus the recipients physical health, mental health and their perception of their general health improved. Regarding the psychopathology variables the levels of Anxiety, Depression and Phobia in the Post COVID-19 phase decreased, while the Somatization score was higher. Lastly, burden of COVID-19 scores in the third phase, significantly decreased.

**Discussion:**

Our study highlights a significant association between mental health and the burden of COVID-19 pandemic in kidney transplant recipients. This study showed, a significant worsening, over time, of some specific symptoms, such as somatization and phobias. However, the results showed that depressive symptoms improved during the study period. Long-term monitoring of kidney transplant recipients therefore remains fundamental. These results confirmed the need to provide integrated multidisciplinary services to adequately address the long-term effects of the COVID-19 pandemic on the mental health of the most vulnerable subjects.

## Introduction

Some longitudinal studies have reported changes in mental health status, both in the general population and in patients with chronic disease, during the two years following the onset of the COVID-19 pandemic.

Bayes-Marin et al. (2023) investigated three components of mental health (psychological stress, personal growth, and loneliness) in 5535 healthy individuals after two years of the COVID-19 pandemic. The results of the study highlighted that secondary or higher education was a factor that negatively influences personal growth, smoking and sleep problems were risk factors for psychological stress, while coping strategies were among the protective factors of the mental health components studied (1). The study by Cuomo et al. (2022) confirmed a strong negative impact on mental health two years after the COVID-19 pandemic in the Italian population. The study considered the point of view of 1281 Italian doctors, who reported an increase in some psychological problems in their patients: agitation, mood and anxiety disorders (2).

Mazza et al. (2022) carried out a longitudinal analysis to evaluate the long-term psychological effects and their impact on the general population, considering three times in particular: the initial period of the lockdown (March 2020), the final period of the lockdown (May 2020) and the two years after the lockdown (July 2022). The differences in the levels of depression, stress, and anxiety in the three periods considered were evaluated. The results of the study showed that most of the participants are now experiencing normal levels of depression, stress, and anxiety, while a smaller percentage of people, at the final period, were experiencing high and extremely high levels of stress, compared to the first two periods (3).

Regarding the psychological impact of COVID-19 on patients with chronic disease, much higher rates of depression and anxiety than the general population were found, and these hampered self-care, adherence to treatment regimens, and caused increased mortality (4, 5). Other studies have highlighted a higher suicidal risk in patients with chronic pathologies compared to normative samples (6–9).

Among patients with chronic disease, there are organ transplant recipients. Transplant recipients must take regular immunosuppressive therapy, which makes these patients more vulnerable and at risk of infections. This, during the COVID-19 pandemic, led to an increased level of alertness and apprehension for them. Some studies investigated the psychological impact of COVID-19 on organ transplant recipients. The study by Merisio et al. (2022) found that resilience may represent a protective factor for liver transplant recipients and liver transplant candidates in mitigating the onset of pandemic-related negative psychological symptoms (10).

Few studies have evaluated the psychological distress of COVID-19 in kidney transplantation and the psychological impact that the COVID-19 pandemic has had on kidney transplant recipients is not yet well understood. The study by Cai et al. (2021) highlighted the presence of symptoms of depression, anxiety, insomnia and post-traumatic stress disorder in 305 organ transplant recipients. Recipients presenting with such symptoms also had lower scores on all dimensions of quality of life (11).

The study by McKay et al. (2021) investigated quality of life, uncertainty, and coping behaviors in 826 solid organ transplant recipients during the COVID-19 pandemic. Furthermore, the following were assessed: previous psychopathologies, perception of the risk of infection and access to treatment. The results of the study showed low levels of uncertainty and acceptance of the disease was the main coping strategy. The impairment of quality of life was, instead, associated with previous psychopathologies, high perception of the risk of infection and limited access to care (12).

Barutcu Atas et al. (2021) found the presence of psychological stress (48.1%), sleep disorders (37.7%), anxiety (23.6%) and depression (44.3%) in 106 kidney transplant recipients. Furthermore, patients who had contracted the COVID-19 infection and patients who had had an episode of rejection reported significantly higher anxiety scores when compared to the others (13).

The study carried out by our research group (14), had explored the impact of COVID-19 and the consequent period of isolation on the perception of health in general, on therapeutic adherence and on the emotional states of kidney transplant recipients, through a questionnaire created *ad hoc* by the authors. Furthermore, the study aimed to evaluate the quality of life and the main psychopathological traits in kidney transplant recipients during the COVID-19 pandemic, through standardized tests.

We valuated 89 kidney transplant recipients and found compromises in emotional state (28.6%) and sleep disturbances (16.1%). Anxiety and phobia symptoms correlated with concerns about their physical health. However, we did not find a negative impact of the COVID-19 pandemic on relational and social aspects, since these were probably well compensated by remote technologies such as video phone calls, Zoom meetings and use of computers.

However, the study had some limitations, among which one of the most important is the short investigation period, during the first phase of COVID-19.

Therefore, the study continued for a further two years after the first wave of the COVID-19 pandemic, in which the same kidney transplant recipients were re-evaluated with the same tools (*ad hoc* questionnaire and standardized tests) in order to assess any changes in long-term mental health. The present study aimed to investigate the change in symptom burden and health-related quality of life in the two years after initial assessment, by outlining the change over time of symptoms at 12 and 24 months of follow-up.

## Materials and methods

### Participants and procedures

We performed a study published in 2021 (phase 1 of COVID-19) (14); of the 89 kidney transplant recipients evaluated in this study, 60 completed the 12 months follow-up (March 2021-June 2021, phase 2 of COVID-19) and 57 completed the 24 months follow-up (March 2022 June 2022, post COVID-19). Only 64% (57 out of 89) of the (small) original sample returned the questionnaires in 2022 for the following reasons: 10 had died due to COVID-19, 10 voluntarily discontinued the study for unreported reasons, 12 did not respond to follow-up phone calls. Moreover, of the total sample, 40 were with COVID-19 infections, 75 were vaccinated.

To not generate variations in the results, we chose to follow up recipients online, as in the first study, to maintain the same evaluation conditions. Questionnaires were sent by mail, of which 36% did not return the documents. Patients were recruited at the Organ Transplant Unit, University Hospital of Catania.

All recipients included in the study were receiving standardized immunosuppressive therapy: Tacrolimus, Mycophenolate Mofetil and Steroids; none of them was taking psychiatric drugs such as antipsychotics and/or antidepressants.

All transplant recipients provided their informed consent to participate in the study. Informed consent was sent by email and participants printed, signed, scanned and sent it back by email. The local ethical committee approved the study procedures in accordance with the Ethical Principles for Medical Research Involving Human Subjects indicated by the 2004 World Medical Association Declaration of Helsinki (15).

### Measures

The *ad hoc* questionnaire on emotional state and psychophysical well-being during COVID-19 included 8 questions: (1) Due to the COVID-19 pandemic how is your overall health? (rated on a 5-point Likert scale: 0 = Excellent, 4 = poor); (2) Has the idea of staying at home, due to COVID-19 isolation, changed your emotional state? (rated on a 5-point Likert scale: 0 = not at all, 4 = very much); (3) If so, how did your emotional state change? How do you feel? (multiple choices possible, such as “I feel worried and anxious”); (4) How worried about your health are you during the COVID-19 pandemic? (rated on a 5-point Likert scale: 0 = Much less than usual, 4 = Much more than usual); (5) Has your sleep quality deteriorated in the last few weeks? (rated on a 5-point Likert scale: 0 = not at all, 4 = very much); (6) In this period of possible COVID-19 contagion, are you afraid to go to the hospital for periodic outpatient checks? (rated on a 5-point Likert scale: 0 = Never, 4 = always); (7) Can you take immunosuppressants in the same way as before COVID-19, according to the doctor’s recommendations? (0 = yes, 1 = no); (8) If you were unable to take immunosuppressive therapy in the same way as before COVID-19, why? (free answer). A total score was calculated (maximum total score = 24), which expresses the “burden related to the pandemic from COVID-19”: the higher the score, the higher the burden caused by COVID-19 (scale reliability: Cronbach’s alpha = 0.71).

The Middlesex Hospital Questionnaire (MHQ) (16) provides a simple and rapid quantification of the main symptoms and relevant traits. Specifically, this questionnaire addresses the rapid quantification of the main “psychological and somatic” symptoms: fluctuating anxiety (ANX), phobic anxiety (PHOB), obsessive-compulsive traits (OBS), somatic symptoms (SOM), depression (DEP), and hysteria (HY). It is made up of 48 items, partly dichotomous (no/yes−0/2), partly assessed on a three-level scale (0–1–2) of frequency or severity of the symptom or behavior explored. The normal reference values, for each of the traits evaluated, are the following: 5.1 (ANX), 2.9 (PHOB), 5.8 (OBS), 3.2 (SOM), 3.3 (DEP), and 7.5 (HY). The MHQ has been found to be a reliable instrument and also valid as a profile measure (16). Also, with regard to our sample of kidney transplant recipients the MHQ showed good reliability (α = 0.87).

The SF-36 (17) was used to assess the health-related quality of life. The Short Form (36) Health Survey is a 36-item, patient-reported survey of patient health. The SF-36 consists of eight scaled scores, which are the weighted sums of the questions in their section. Each scale is directly transformed into a 0–100 scale on the assumption that each question carries equal weight. The lower the score the more disability. The higher the score the less disability. The eight sections are: vitality (VT), physical functioning (PF), bodily pain (BP), general health perceptions (GH), physical role functioning (PR), emotional role functioning (ER), social role functioning (SR), and mental health (MH). The questionnaire also provides two summary measures, one relating to Physical Component Summary (PCS) and one relating to Mental Component Summary (MCS). Even for these two scores, the higher the values, the better the perceived physical and mental health.

The validity and reliability for SF 36 have been confirmed in patients with chronic kidney disease and in kidney transplant recipients (17). Also, with regard to our sample of hemodialysis patients, the SF-36 showed good reliability (α = 0.92).

### Statistical analysis

Statistical analyses were conducted using SPSS 29. The description of the sample was analyzed with descriptive statistics. Line graphs show the means of the variables (burden C-19, GH, MHI, PHI, ANX, DEP, SOM and PHOB), through the stages. The Spearman correlation was used for analysis between burden C-19, MHQ, SF-36 and some demographic characteristics of the sample in the three COVID-19 stages. All significance levels reported are two-tailed. A repeated measure ANCOVA was conducted to determine a statistically significant difference between the three groups (first phase of COVID -19, second phase of COVID-19 and post COVID -19) on burden C-19 and controlling for the MHI, PHI, ANX, DEP, SOM, PHOB and GH covariates.

## Results

### Participants

Of the 89 recipients, 43% were men 57% were women. Patient ages ranged from 20 to 75 years old, most of the sample is found in the age group 51-60 years Regarding education, most of the sample finished high school. Most of the patients are employed. The majority of the sample was diagnosed with kidney disease in a range between 21-30 years ago (**Table 1**). Of the 89 participants, 60 completed the 12-month follow-up and 57completed the 24-month follow-up. The number of participants dropped to 64% (n = 57; 63% men; M_age_ = 51.5; *SD*_age_ = 11.66). Other demographic variables of the patients (N=57) who completed 24 months follow- up, frequency and proportion are shown in **Table 1**.

**Table 1:** Demographic characteristics of patients included in the study (24 months follow-up N=57).

	Levels	Frequency	%
**Sex**	Female	52	58%
	Male	37	42%
**Age**	20-30	8	9%
	31-40	11	12%
	41-50	21	24%
	51-60	35	39%
	61-75	14	16%
**Education**	Primary school	6	7%
	Secondary school	12	13%
	High school	56	63%
	College, University or post-doctoral degree	15	17%
**Profession**	Student	15	17%
	Housewife	15	17%
	Unemployed	8	9%
	Retired	8	9%
	Employed	43	48%
**Marital status**	Unmarried (never married)	16	18%
	Married	51	57%
	Widow	13	15%
	Divorced	9	10%
**Transplant history**	Pre-emptive kidney transplant	2	2%
	Two or more transplants	4	4%
	Living kidney transplant	31	35%
	Kidney transplant	52	59%
**Kidney failure**	Autoimmune disease (LES)	10	11%
	Polycystic kidney	34	38%
	Patient unable to recall the cause of kidney failure	18	20%
	Glomerulonephritis	14	16%
	Congenital absence of one kidney	7	8%
	Diabetes	6	7%
**Years since diagnosis of kidney disease**	5-12	12	14%
	13-20	18	20%
	21-30	33	37%
	>31	26	29%
**Kidney rejection** **(Pre COVID-19)**	No	75	84%
	Yes	14	16%
**Years since transplant procedure**	1-10	44	49%
	11-21	20	23%

**Table 2** shows the mean comparison of GH, PHI, MHI, ANX, DEP, SOM, PHOB and burden C-19 between phase 1 of COVID-19, phase 2 of COVID-19 and phase 3 of COVID-19. Compared to the first and second phase of COVID-19, the mean score of GH, PHI and MHI variables were higher in the Post COVID-19 phase (M-GH= 52, SD-GH=20; M-PHI= 45, SD-PHI= 11,5; M-MHI= 47, SD-MHI= 11); thus the recipients physical health, mental health and their perception of their general health had improved. Regarding the MHQ variables the levels of Anxiety, Depression and Phobia in phase 3 of COVID-19 decreased, while the Somatization score was higher (M-ANX= 5, SD-ANX=3; M-DEP= 4, SD-DEP=2,5; M-PHOB=4, SD-PHOB= 2,8; M-SOM= 7, SD-SOM= 3,5). Lastly burden COVID-19 scores in the third phase, significantly decreased (M-burden COVID-19 = 6,7, SD-burden COVID-19 = 2,5). Others mean score not showed in the figure for SF36 are for phase 1: PF= 73; PR= 65,3; BP=70; VT= 56; SR= 71; ER= 62; MH= 63; while for phase 2 are: PF= 60; PR= 61; BP= 62; VT= 51; SR= 58; ER= 72; MH= 71; lastly for phase 3: PF= 78; PR= 63; BP=70; VT= 60; SR= 65; ER= 59; MH= 73. Also, MHQ OBS and HY score did not report a difference through the phases (M-OBS = 6,1; SD= 3,4; M-HY = 3,4; SD=2,8). The Burden of COVID-19 total score has decreased from phase 1 to phase 2 of 33.33%, and 16.25% from phase 2 to phase 3 and from phase 1 to phase 3 of 44.17%.

**Table 2:** Mean and SD scores comparison between Phase 1, Phase 2 and Phase 3.

Variables	Phase 1		Phase 2		Phase 3	
**N**= 57	M	SD	M	SD	M	SD
**GH**	50	18.56	51	19.28	52	20
**PHI**	45	9.83	42	10.2	45	11.5
**MHI**	43	10.31	46	10.83	47	11
**ANX**	6	3.21	5.50	3.12	5.10	3
**DEP**	6	2.7	5.40	2.51	4.00	2.5
**SOM**	4.60	3.01	4.88	3.22	6.83	3.5
**PHOB**	5.20	2.51	4.50	3.31	4.22	2.8
**BURDEN C-19**	12.00	3.82	8.00	3.2	6.70	2.5

ANX, Anxiety; DEP, Depression, SOM, Somatization; PHOB, Phobia; PHI, Physical Health Index; MHI, Mental Health Index; GH, General Health Perceptions; Burden C-19, Burden COVID-19; M, Mean score; SD, standard deviation.

**Table 3** shows the Spearman correlation coefficient (r) performed in phase 1 of COVID-19, phase 2 of COVID-19 and phase 3 of COVID-19, between demographic variables of the recipients, the burden of COVID-19, GH, MHI and PHI from the SF-36 questionnaire, and the MHQ. There were significant positive correlations in the first phase of C-19, between burden C-19 and some demographic variables: Age (r = 0.830, p< 0.05), level of education (r = 0.441, p< 0.05), with Years since transplantation (r = 0.763, p< 0.05). Regarding the items form the MHQ there were also significant positive correlations between OBS with burden C-19 (r = 0.180, p< 0.05) and HY with burden C-19 (r = 0.361, p< 0.05). Burden C-19 also negatively correlated with PHI (r = - 0,380, p< 0.01) and with MHI (r =-0,408, p< 0.01). In phase 2 of COVID-19, there were different significant correlations between burden C-19: negative correlation with Kidney Rejection (r = - 0,368, p< 0.01). Regarding SF36, burden C-19 negative correlates with GH (r = - 0,423, p< 0.05), and with MHI (r = -0,263, p< 0.05). Regarding the main symptoms and relevant traits measured with the MHQ, burden C-19 positive correlates with SOM (r = 0,269, p< 0.01), and with PHOB (r = 0,544, p< 0,01). Lastly, the phase 3 of COVID-19 shows that burden C-19 correlates positively with demographic variable Sex (r= 0,254, p< 0.05), the MHQ item DEP (r= 0,385, p< 0.01), and SOM (r =0,344, p< 0.01), and negatively with GH (r= -0,423, p< 0.01). Other significant correlations are reported in **Table 2**.

**Table 3:** Spearman correlation coefficient (r) of the 3 phases between demographic characteristics and burden of COVID-19, MHQ and some items from SF36.

Phase 1 of C-19											
	GH	MH	PHI	MHI	ANX	DEP	SOM	PHOB	OBS	HY	Burden C-19
**Sex**	.948*	.006	-.119	-.147	.213	.832*	.029	.001	.303	.499	.106
**Age**	.581	.047	-.371^**^	.264^**^	.114	.585*	.293	.321	.547*	.108	.830*
**Education**	.281	.990*	.390^**^	-.134	.034	.264	-.481*	.323	.234	.012	.441^*^
**Profession**	.079	-.048	.215^*^	-.142	.050	-.003	-.087	-.024	.176	-.072	-.042
**Transplant history**	.142	.372^**^	.068	.364^**^	-.046	-.101	.020	.023	.014	.071	-.045
**Years since diagnosis**	-.072	-.057	-.132	-.068	-.049	.102	.224^*^	.198^*^	-.054	-.184	.009
**Years from Transplantation**	.647*	.459*	-.006	-.071	.047	.316*	.448*	.168	.160	.399*	.763*
**Kidney failure**	-.010	.090	-.084	.020	-.194^*^	-.053	-.003	.019	-.251^**^	-.104	-.045
**Kidney rejection**	.115	.046	.189^*^	-.032	.039	-.139	.012	-.041	.210^*^	.076	-.085
**Burden C-19**	.005	.000	-.380**	-.408^**^	.013	.006	.000	.046	.180*	.361*	
Phase 2 of C-19											
	GH	MH	PHI	MHI	ANX	DEP	SOM	PHOB	OBS	HY	Burden C-19
**Sex**	.114	-.064	.056	-.131	.129	.038	.135	.400^**^	-.017	-.223	.227
**Age**	.025	-.078	-.078	.001	-.036	.109	.086	.199	.025	-.036	.033
**Education**	-.175	-.093	.033	-.195	.152	.112	-.021	-.115	.121	.352^**^	.032
**Profession**	.073	-.245	-.056	-.128	.135	.252	.096	.209	.094	.073	-.052
**Transplant history**	.131	.222	.210	.006	.037	.079	-.131	-.071	.095	.124	-.158
**Years since diagnosis**	-.080	-.074	-.092	.080	-.059	-.135	-.109	.174	-.119	.094	.136
**Years from Transplantation**	-.051	.012	-.169	-.018	.049	-.008	.246	.228	-.048	-.153	-.021
**Kidney failure**	.204	.229	.180	.000	-.201	-.212	-.090	-.374^**^	-.246	-.204	-.079
**Kidney rejection**	.213	.091	-.022	.018	-.028	-.010	-.123	-.178	-.005	.027	-.368^**^
**Burden C-19**	-.423^**^	-.263^*^	-.226	-.142	.132	.004	.269^*^	.544^**^	-.188	-.043	
Phase 3 of C-19											
	GH	MH	PHI	MHI	ANX	DEP	SOM	PHOB	OBS	HY	Burden C-19
**Sex**	-.009	-.149	-.015	-.045	.161	.306^*^	.055	.090	.105	-.143	.254^*^
**Age**	-.024	.052	-.083	.182	-.100	.185	-.137	.063	.092	-.122	.106
**Education**	-.046	-.093	.001	-.062	.114	-.174	-.024	.016	-.071	.226	-.052
**Profession**	-.053	.045	-.168	.105	-.096	-.155	-.208	-.031	-.085	-.107	.035
**Transplant history**	.049	.242	.243	.124	-.014	-.046	.088	-.105	-.154	-.021	-.088
**Years since diagnosis**	-.140	-.121	-.217	-.019	.139	.079	-.063	.207	.080	-.095	-.070
**Years from transplantation**	-.006	.105	-.049	.025	.073	.058	.051	-.139	.022	.041	.221
**Kidney failure**	-.090	.116	-.087	.094	.055	.069	.017	.022	.008	.013	.216
**Kidney rejection**	.077	.064	-.068	-.013	-.044	-.206	-.192	-.196	-.016	-.075	-.095
**Burden C-19**	-.423^**^	-.184	-.209	-.177	.175	.385^**^	.344^**^	-.094	.021	.209	

C-19, COVID-19; MHQ, Middlesex Hospital Questionnaire; ANX, Anxiety; DEP, Depression, SOM, Somatization; PHOB, Phobia; OBS, Obsession; HY, Hysteria; SF-36, Short form (36) Health Survey; PHI, Physical Health Index; MHI, Mental Health Index; GH, General Health Perceptions; MH, Mental Health; E, Education; Years from transplantation; Burden C-19, Burden COVID-19.

Sex: Male= 0, Female= 1.

**. Significance 0.01 (two-tailed).

*. Significance 0.05 (two-tailed).

A repeated measure ANCOVA was conducted (**Table 4**) to determine a statistically significant difference between the three stages (first phase of COVID -19, second phase of COVID-19 and phase 3 of COVID -19) on burden COVID-19 and controlling for the MHI, PHI, ANX, DEP, SOM, PHOB and GH covariates. **Table 4** shows ANCOVA results also with the Difference Mean scores for each phase. There’s a significant effect of groups on burden COVID-19 after controlling for the F-GH (1,501), F-MHI (1, 228) = 20,1, F-PHI (1,174), F-ANX (1,297,4), F-DEP (1, 333), F-SOM (1,335), F-PHOB (1, 336,4) p< 0.001. There was also a significant effect of groups on levels of burden of COVID-19 after controlling for the effect of the covariates. Planned contrasts revealed that in the first phase of COVID-19, burden C-19 significantly increased compared to having a control, t-PHI(501)=9,5,t-MHI(228)= 9,07, t-PHI(174)=9,09, t-ANX(297,4)=9, F-DEP(333)=8,11, F-SOM(335)=10,7, t-PHOB(336,4)=9,1, all covariates p< 0.001. There’s a significantly difference compared to the second phase of COVID-19 t-PHI(501)=2,8, p=0.005, t-MHI(228)= 2,9, p=0.004, t-PHI(174)=2,5, p= 0.013, t-ANX(297,4)=2,6, p=0.011, F-DEP(333)=1,9, p=0.060, F-SOM(335)=4, p<0.001, t-PHOB(336,4)=2,5, p=0.012. In phase 1, the mean of burden COVID-19 between controls is significant (p< 0.001), while in phase 2 it is not significant except for the variable SOM. Considering the averages of the three phases responsible for the contribution of the covariates GH, MHI, PHI, ANX, SOM and PHOB the comparison indicates that the first phase is statistically different from the second and third phase, and the second phase is statistically different from the third phase. Regarding covariate DEP, the first phase is statistically different from the second and third phase, but the second phase is similar to the third phase (difference mean scores are shown in **Table 3**).

**Table 4:** Repeated measure ANCOVA.

Covariates				Intercept	Groups		Phase 1 (N=89)	Phase 2 (N=60)	Phase 3 (N=57)	MD PHASE 1	MD Phase 2	MD Phase 3
	F	P	t	f/p	F	P	t/p	t/p	t/p	Phase2/Phase 3	Phase1/Phase 3	Phase1/Phase2
**GH**	23,5	<.001	-4.9	501/<.001	50.4	<.001	9.5/<.001	2.8/.005	0^a^	3.2*/4.8*	-3.2*/1.6*	-4.8*/-1.6*
**MHI**	20,1	<.001	-4.5	228.2/<.001	44.8	<.001	9.07/<.001	2.9/.004	.	2.9*/4.6*	-2.9*/1.7*	-4.6*/-1.7*
**PHI**	6,5	0.012	-2.5	174/<.001	46.5	<.001	9.09/<.001	2.5/.013	.	3.2*/4.32*	-3.2*/1.14*	-4.32*/-1.14*
**ANX**	24,4	<.001	5	297.4/<.001	45	<.001	9/<.001	2.6/.011	.	3.04*/4.5*	-3.04*/1.5*	-4.5*/-1.5*
**DEP**	9,2	.003	3.04	332.6/<.001	39.6	<.001	8.11/<.001	1.9/.060	.	3.2*/4.32*	-3.2*/1.14	-4.3*/-1.14
**SOM**	29	<.001	5.4	334.6/<.001	61	<.001	10.7/<.001	4/<.001	.	3.2*/5.5*	-3.2*/2.2*	-5.5*/-2.2*
**PHOB**	32,6	<.001	5.7	336.4/<.001	46.7	<.001	9.1/<.001	2.5/.012	.	3.1*/4.5*	-3.1*/1.41*	-4.5*/1.41*

ANCOVA: Dependent variable burden covid-19 with GH, MHI, PHI, ANX, DEP, SOM, PHOB as a covariates revealed significant group differences.

Phase 3 of COVID-19 phase parameter “a.” is set to zero because it is redundant.

MD, Mean Difference.

## Discussion and conclusions

The present study aimed to investigate the psychopathological impact of the COVID-19 pandemic on kidney transplant recipients two years after the first wave of the pandemic by outlining the trajectory of symptoms at 12 and 24 months of follow-up. This study confirmed the strong mental health impact of the last two years of the COVID-19 pandemic in kidney transplant recipients. A pandemic, as a traumatic event, can lead to strong emotional discomfort generating dysfunctional emotional reactions. The increase in mental symptoms due to the COVID-19 pandemic particularly affected fragile subjects with compromised immune systems such as organ transplant recipients.

This study showed a significant worsening, over time, of some specific symptoms, such as somatization, even if other dimensions of mental health, such as depression, gradually reduced.

Regarding mental health, the “somatizations” variable of the MHQ, as already mentioned in the previous study for the first evaluation time, worsened over time in the second phase of COVID-19, especially in third phase. These data relating to the somatic symptom represents the result of a condition in which the psychological stress from COVID-19 negatively influenced physiological (somatic) functioning to the point of causing suffering. Psychological well-being was strongly influenced by the psychological stress that transplant recipients experienced during the difficult period of the COVID-19 pandemic, the effects of which continue to persist to this day. These considerations are supported by the recent literature.

The study by Thangaraju et al. (2022) assessed the health status and psychosocial impact of COVID-19 in 325 kidney transplant recipients using a survey questionnaire. Anxiety, depression, and psychological stress were assessed using standardized instruments. A prevalence of psychological stress of 12.8% was found in kidney transplant recipients (18). In the study by Gundogmus et al. (2023) kidney transplant recipients experienced high levels of psychological distress as well as the loss of caregivers and social support during the COVID-19 pandemic (19).

The symptom “Phobia”, already evident in the first phase of this study, tends to decrease in the second and third phases. With the end of the obligation to isolate and the condition of protective isolation no longer present, phobic thoughts such as contamination phobia and fear of contagion became less evident in transplant recipients (20–22),.

Regarding depression, the results of this study highlighted a change in this symptom in the three evaluation phases. In particular, if depression was present in the first two periods, in the third period it was reduced, reaching a value slightly above the norm. These data are probably related to the fact that, with the end of isolation, patients felt more confident in resuming their interpersonal relationships, gradually returning to a “normal” life. The current literature supports these data. In the meta-analysis of longitudinal studies by Robinson et al. (2022) an acute increase in psychopathological symptoms was found in the early stages of the COVID-19 pandemic, symptoms which significantly reduced over time and gradually returned to pre-pandemic conditions (21).

In our study, interesting associations also emerged between the Burden of COVID-19 and some demographic characteristics of the sample, in the three evaluation phases. In particular, in the first phase, as age, years of education and years since transplant increase, the Burden of COVID-19 increases. In both the second and third phases, the Burden of COVID-19 increases as somatization traits increase. In the third phase, the Burden of COVID-19 increases as rejection decreases. This last data is probably related to the fact that kidney transplant recipients who have not experienced rejection are more afraid of losing the transplanted kidney, especially during the phases of COVID-19, while those who have already experienced it appear more resigned. Finally, an interesting association has emerged between the female sex and the Burden of COVID-19: women seem to be more affected by the burden of COVID-19. This data is consistent with the literature on the topic, in which women appear to be more predisposed and vulnerable to adverse events, and their mental health has been particularly affected during the COVID-19 pandemic (23).

In relation to the results, we want to underline the main concept that transplant recipients need to be monitored adequately and promptly, and this, during the pandemic, may have undergone alterations due to the COVID-19 emergencies that our healthcare system had to face.

Long-term monitoring of kidney transplant recipients, therefore, remains fundamental, as psychopathological symptoms can also undergo changes based on more or less stressful living conditions that are experienced during one’s life.

These results confirmed the need to provide integrated multidisciplinary services to adequately address the long-term effects of the COVID-19 pandemic on the mental health of the most vulnerable subjects (24–27). The results of our study are also useful because they can be used in future epidemics/pandemics.

However, this study has limitations that should be noted when interpreting the main findings. First, it is an observational study, which precludes causal inferences among the variables analyzed.

Second, the patients could contract COVID-19 during the follow up, this could cause interferences with the performed evaluations. Third, the size of the sample is small furthermore there was a significant loss of participants after the first phase, which could have randomly influenced the results of the study and there may be a bias due to the online compilation of the questionnaires: the self-reported levels of psychological impact may not always be aligned with objective assessments by mental health professionals. Finally, further longitudinal studies with large sample sizes need to validate the present findings.

## Data availability statement

The original contributions presented in the study are included in the article/supplementary material. Further inquiries can be directed to the corresponding author.

## Ethics statement

The studies involving humans were approved by Ethics Committee Catania 1 University Hospital Policlinico G. Rodolico-San Marco, Catania, Italy. The studies were conducted in accordance with the local legislation and institutional requirements. The participants provided their written informed consent to participate in this study.

## Author contributions

CD: Writing – original draft, Funding acquisition, Conceptualization. MP: Writing – original draft, Conceptualization. MV: Writing – review & editing, Supervision. NB: Writing – original draft, Formal analysis, Data curation. PC: Writing – review & editing, Data curation. BE: Writing – review & editing, Supervision. GL: Writing – review & editing, Data curation. AG: Writing – review & editing, Data curation. PV: Writing – review & editing, Validation, Supervision.
